# Supplementary material for: Down-regulation of TORC2-Ypk1 signaling promotes MAPK-independent survival under hyperosmotic stress
Source: eLife. 2015 Aug 14;4:e09336. doi: 10.7554/eLife.09336 (PMC4552222; doi:10.7554/eLife.09336)
Supplement: Supplementary file 1. — Yeast strains used in this study. DOI: http://dx.doi.org/10.7554/eLife.09336.011 [file elife09336s001.docx]

**Table 1** Yeast strains used in this study.

| **Strain** | **Genotype** | **Source/reference** |
| --- | --- | --- |
| BY4741 | *MAT***a** *his3∆1 leu2∆0 met15∆0 ura3∆0* | Research Genetics, Inc. |
| BY4742 | *MAT***α** *his3∆1 leu2∆0 lys2∆0 ura3∆0* | Research Genetics, Inc. |
| JTY6142 | BY4741 *ypk1∆::*KanMX4 | Research Genetics, Inc. |
| yAM135-A | BY4741 Ypk1(L424A)::*URA3-* *ypk2∆::* KanMX4 | (Muir et al., 2014) |
| yAM181-A | BY4742 *fps1∆::*natNT2 | This study |
| yAM271-A | BY4742 Fps1-3xFLAG::*URA3-* | This study |
| yAM272-A | BY4742 Fps1(S181A S185A S570A)-3xFLAG::*URA3-* | This study |
| yAM275 | BY4742 *LYS2^+^* Fps1-3xFLAG::*URA3 hog1∆::*KanMX | This study |
| yAM278 | BY4742 Fps1(S181A S185A S570A)-3xFLAG::*URA3 hog1∆::*KanMX | This study |
| yAM281 | BY4742 Ypk1(L424A)::*URA3-* *ypk2∆::* KanMX4 Fps1-3xFLAG::*URA3* | This study |
| yAM284-A | BY4742 Ypk1(L424A)::*URA3-* *ypk2∆::* KanMX4 Fps1(S181A S185A S570A)-3xFLAG::*URA3* | This study |
| yAM291-A | BY4742 Fps1(S570A)-3xFLAG::*URA3 hog1∆::*KanMX | This study |
| yAM301-A | BY4742 Fps1(S181A S185A)-3xFLAG::*URA3* | This study |
| yAM307-A | BY4742 Fps1(Δ544-581)-3xFLAG::*URA3* | This study |
| yAM308-A | BY4742 Fps1(I218A V220A)-3xFLAG::*URA3* | This study |
| yAM309-A | BY4742 Fps1(S181A S185A I218A V220A S570A)-3xFLAG::*URA3* | This study |
| yAM310-A | BY4742 Fps1(T147A)-3xFLAG::*URA3* | This study |
| yAM315 | BY4741 Rgc2(S344A T808A S948A S75A S827A S1021A S1035A)-3xHA:: Hyg^r^ | This study |
| yAM318 | BY4741 Fps1(S181A S185A S570A)-3xFLAG::*URA3* Rgc2(S344A T808A S948A S75A S827A S1021A S1035A)-3xHA:: Hyg^r^ | This study |
| yGT21 | BY4742 Fps1-3xFLAG::*URA3* | This study |
| yGT22 | BY4742 Fps1(S181A S185A S570A)-3xFLAG::*URA3* | This study |
| yGT24 | BY4742 Fps1(S570A)-3xFLAG::*URA3* | This study |
| YJP544 | BY4741 *hog1∆::*KanMX | Jesse Patterson, this lab |
| yKL5 | BY4741 Tor2(L2178A)::Hph (Hyg^R^) | (Muir et al., 2014) |
| JTY5468 | BY4741 *tor2-29::*KanMX | (Li et al., 2011) |
| JTY5574 | BY4741 *cna1∆::KanMX cna2∆::KanMX* | Gift of Aaron Goldman (M.S. Cyert Lab, Stanford Univ.) |
| JTY5537 | BY4742 *pbs2∆::*KanMX | Research Genetics, Inc. |
| JTY5538 | BY4742 *ssk2∆::*KanMX | Research Genetics, Inc. |
| JTY5539 | BY4742 *ssk22∆::*KanMX | Research Genetics, Inc. |
| JTY5540 | BY4742 *sho1∆::*KanMX | Research Genetics, Inc. |
| JTY5541 | BY4743 *ssk1∆::*KanMX*/ssk1∆::*KanMX | Research Genetics, Inc. |
| DL3188 | BY4742 *rgc1∆::*KanMX *rgc2∆::*KanMX | (Beese et al., 2009) |
