# Supplementary material for: Down-regulation of TORC2-Ypk1 signaling promotes MAPK-independent survival under hyperosmotic stress
Source: eLife. 2015 Aug 14;4:e09336. doi: 10.7554/eLife.09336 (PMC4552222; doi:10.7554/eLife.09336)
Supplement: Supplementary file 2. — Plasmids used in this study. DOI: http://dx.doi.org/10.7554/eLife.09336.012 [file elife09336s002.docx]

**Table 2.** Plasmids used in this study.

| **Plasmid** | **Description** | **Source/reference** |
| --- | --- | --- |
| pRS315 | *CEN*, *LEU2*, vector | (Sikorski and Hieter, 1989) |
| pRS316 | *CEN*, *URA3*, vector | (Sikorski and Hieter, 1989) |
| pAX238 | pRS316 P*_GPT2_*-Gpt2-3xFLAG | This study |
| pAX244 | pRS316 P*_GPT2_*-Gpt2(S649A S650A S651A)-3xFLAG | This study |
| pAX274 | pRS316 P*_FPS1_*-Fps1-3xFLAG | This study |
| pAX275 | pRS316 P*_FPS1_*-Fps1(S181A S185A S570A)-3xFLAG | This study |
| pAX290 | pRS316 P*_FPS1_*-Fps1-GFP | This study |
| pAX293 | pRS316 P*_FPS1_*-Fps1(S570A)-GFP | This study |
| pAX294 | pRS316 P*_FPS1_*-Fps1(S181A S185A)-GFP | This study |
| pAX295 | pRS316 P*_FPS1_*-Fps1(S181A S185A S570A)-GFP | This study |
| pAX302 | pRS315 P*_MET25_*-Fps1-3xFLAG | This study |
| pAX303 | pRS315 P*_MET25_*-Fps1(S181A S185A S570A)-3xFLAG | This study |
| pFR252 | pRS315 P*_YPK1_*-Ypk1(S51A S57A S71A T504A S644A S653A T662A)-myc | This study |
| p3151 | pRS316 P*_MET25_*-Rgc2-3xHA | (Lee et al., 2013) |
| pPL215 | p416 P*_MET25_*-Ypk1-3xHA | (Niles et al., 2012) |
| pGEX6P-1 | GST tag, bacterial expression vector | GE Healthcare, Inc. |
| pBT7 | pGEX6P-1 Fps1(531-669) | (Muir et al., 2014) |
| pAX135 | pGEX6P-1 Fps1(531-669)(S570A) | This study |
